# Bovine leukemia virus and dairy youngstock: a narrative review of early-life transmission and its consequences for whole-herd control

**DOI:** 10.3389/fvets.2026.1911190

**Published:** 2026-10-23

**Authors:** Madison E. Sokacz, Bo Norby, Kelly R. B. Sporer, Daniel D. Buskirk, Tasia M. Taxis

**Affiliations:** 1 Department of Animal Science, Michigan State University, East Lansing, MI, United States; 2 Department of Large Animal Clinical Sciences, Michigan State University, East Lansing, MI, United States; 3 Cornerstone Ag Management, LLC, Hastings, MI, United States

**Keywords:** biosecurity, bovine leukemia virus, dairy farms, neonatal, transmission risk, youngstock periods

## Abstract

Bovine Leukemia Virus (BLV) is a retrovirus that causes a lifelong infection in cattle through the integration of proviral DNA into the host genome, creating immune dysfunction. Youngstock, females born and raised on dairy farms with the purpose of entering the milking herd, are exposed to BLV through both vertical and horizontal transmission risks. As youngstock progress through distinct management development periods (fetal and neonatal, preweaning, and post-weaning), each presents unique exposure risks that require different management strategies to aid in the mitigation of BLV. The failure to address BLV infection in youngstock allows infected replacement animals to enter the milking herd, undermining any mature cow-focused control strategies. The economic impacts may also be compounded by reduced return on investment in youngstock. Targeted interventions during the youngstock periods, informed by diagnostic screening for BLV and improved management strategies, are essential to disrupt the cycle of infected youngstock entering the milking herd. Incorporating BLV control measures with the entire herd offers a more sustainable approach to reducing BLV prevalence and limiting its long-term effects on animal health and profitability.

## 1 Introduction

The earliest account of bovine leukemia description was in 1871 in Lithuania where an enlarged spleen with nodules was described during necropsy (1). While these clinical signs were suggestive of bovine leukosis, the causative agent was not identified until nearly 100 years later (2). By 1985, the virus, now known as Bovine Leukemia Virus (BLV), was successfully sequenced and classified in the genus *deltaretrovirus* within the family *Retroviridae* (3). This classification placed BLV with other retroviruses, such as human T-lymphotropic virus (HTLV-1 and−2) and simian T-lymphotropic virus (STLV-1,−2,−3), which are characterized by the ability to reverse transcribe RNA into DNA provirus (4).

There are four general stages of BLV infection: initial infection, aleukemic stage, persistent lymphocytosis (PL) stage, and tumor development (5). Once BLV is transmitted

to an animal the initial infection stage begins. The BLV virion targets the B-lymphocytes (6) and utilizes reverse transcriptase to integrate its DNA into the host genome. This DNA integration into the host genome characterizes BLV as a chronic infection (7). The infected host cells then go on to replicate, which in turn replicates the proviral DNA. Following integration, proteins within the BLV proviral genome will activate cellular oncogenes, which in turn starts the dysregulation of the animal's immune system (8). This initial infection stage is asymptomatic.

Following initial infection, most infected animals will enter the aleukemic stage in which they are infected but remain subclinical and do not show signs of hematological abnormalities (9). Despite the lack of clinical signs or laboratory abnormalities, the BLV provirus begins to gradually proliferate by clonal expansion during the aleukemic stage (10). During the aleukemic stage the serological and molecular detection of BLV is possible and the aleukemic animal can infect other animals (11).

The next stage, known as persistent lymphocytosis, is characterized by an abnormal and sustained expansion of B-lymphocytes (10). In this stage, BLV multiplies exponentially within B-lymphocytes (10, 12). While animals may still appear normal clinically, PL indicates an ongoing immune response and can be identified through hematological assays. The immune dysregulation experienced in this stage is linked to increased susceptibility to secondary infections and reduced vaccination efficacy (10, 13).

The final stage of BLV infection may culminate in lymphomas or tumor development. Lymphomas are defined as the formation of solid tumors when infected lymphocytes undergo malignant mutations (11). These malignant developments can occur in various areas throughout the body, including the heart, abomasum, uterus, lymph nodes, eye socket, or spinal canal (7, 10, 12, 14). Clinical signs can vary depending on the tumor location, but may include weight loss, decreased appetite, neurological dysfunction, or sudden death (10, 15). The lymphosarcoma stage is subsequently fatal through culling or death (10).

Following the identification of BLV, various geographical regions have experienced different levels of urgency and desire to work toward control and eradication. Within the United States, it is estimated that 94% of dairy herds are infected with BLV, and the within-herd prevalence is 46% (16). Despite the frequency with which BLV occurs, the impact on the immune system, and the classification as a notifiable disease by the World Organization for Animal Health (17), there are no national standards for testing, mitigation strategies, or governmental incentives for BLV eradication within the United States. Prevalence of BLV and control efforts vary considerably outside the United States as well; some countries and regions have implemented eradication programs and report substantially lower within-herd prevalence, while comparable youngstock-specific data from many regions remain limited. The present review focuses primarily on the U.S. context, described further below.

## 2 Diagnostics

The first cases of BLV were discovered because of the development of lymphomas in cattle and lymphomas were considered to be the first diagnostic tool as it was pathognomonic for the disease (18). The development of lymphomas is the final stage of BLV progression. These lymphomas would primarily develop within peripheral lymph nodes. The detection of lymphomas can occur through palpation of lymph nodes; the most common detection occurs post-mortem and results in the condemnation of the entire carcass (19). Following the discovery of tumor development, an association was made with accompanying increased lymphocyte counts (15).

The development and use of serological assays that identify immune response through the detection of BLV-specific antibodies became the most common method for diagnosing (17). Serological assays used for the detection of anti-BLV antibodies consist of radioimmunoassay (RIA), agar-gel immunodiffusion (AGID), and enzyme-linked immunosorbent assays (ELISA). Early studies utilized RIA (20–23), but the assay was phased out of use due to the safety requirements for working with radioactivity. Researchers and clinicians then began to utilize the AGID assay for the detection of BLV specific antibodies (24–26). The use of AGID is labor intensive due to the purification process of the BLV antigen and the molding of the agarose gels as well as the extensive process time required (27). Only serum can be utilized for AGID, whereas the ELISA can be utilized on a wider range of samples that includes serum, milk, and colostrum. The BLV ELISA optical density (OD) (S/P ratio) outcome is continuous. The OD of the sample is often reported with established thresholds that determine categorical results, such as negative, suspect, and positive, depending on the specific kit. Detection of BLV antibodies by serum and milk ELISA has been reported to have as high as 100% agreement (28). A more recent comparison indicates that when compared to a serum ELISA, milk ELISA had 86% and 100%, respectively (29).

While the serological detection of BLV infection is the most frequently used diagnostic method, viral DNA can be detected directly through using polymerase chain reaction (PCR). The PCR may be utilized on different types of samples such as semen, milk, mucosal samples, and whole blood. Detection by PCR can either be qualitative or quantitative. The qualitative detection is usually performed through standard endpoint PCR with agarose gels to indicate either presence or absence of BLV within the sample. A known limit for PCR is the potential for false negatives, likely due to the ability for BLV to persist at low levels and to sequester within organs (30). To combat this potential limitation, nested PCR have been used due to their use of two sets of primers utilized in two consecutive PCR assays (31, 32). However, as nested PCR increase the sensitivity of the assay, the specificity is decreased, which increases the likelihood of false positives (17, 33). The quantitative PCR (qPCR) strengthens the diagnostic method by determining the number of proviral copies in the sample in respect to an endogenous gene or quantity of DNA (34). This allows for the quantification of detected infections rather than binary detection. Results are commonly stated as proviral load (PVL), the ratio of BLV genome copies to β-actin copies. The number of BLV copies per unit of blood and PVL are often used to interpret the infectivity of an animal (8). However, a commonly known limitation to the qPCR is the ability to accurately quantify the number of proviral copies. Studies comparing the various qPCR assays have observed variability in detecting BLV provirus (35, 36).

## 3 Transmission

Transmission of BLV occurs through both horizontal and vertical routes. Horizontal transmission refers to the spread of the virus between animals, through direct contact with infected bodily fluids; blood, milk, saliva, or nasal secretions containing infected lymphocytes (37–40). Vertical transmission is the transfer of the virus from an infected dam to her calf, during pregnancy through the placenta, during birth, or after birth through colostrum or milk (38, 41–43). However, determining when vertical infections happened is difficult due to the delay in detecting the virus after infection. This lag is estimated to be 24 and 36 days post infection for qPCR and ELISA assays, respectively (40). Additionally, youngstock acquire passive immunity through colostrum, which can interfere with immunologic detection of BLV (e.g., ELISA), as test results may reflect maternally derived antibodies rather than a calf's true infection status.

## 4 Impact on youngstock and dairies

As infected youngstock mature and enter the milking herd, the youngstock then contribute to the milking herd BLV prevalence and it increases the risk of infection of cows that are BLV negative. Most research and on-farm management efforts have focused on the management of BLV in the milking herd, because the mature milking cows generally comprise the profit generating portion of a farm. Studies have shown that BLV management, such as segregation or culling of infected lactating animals can reduce within-herd BLV prevalence (8, 44–46). While the proper management of BLV in the lactating cows is crucial to mitigating the spread and impact of BLV, it is also critical to manage BLV in youngstock before they mature into lactating animals, to avoid creating a cyclical pattern of BLV prevalence being driven by infected youngstock entering the milking herd (Figure 1).

**Figure 1:**
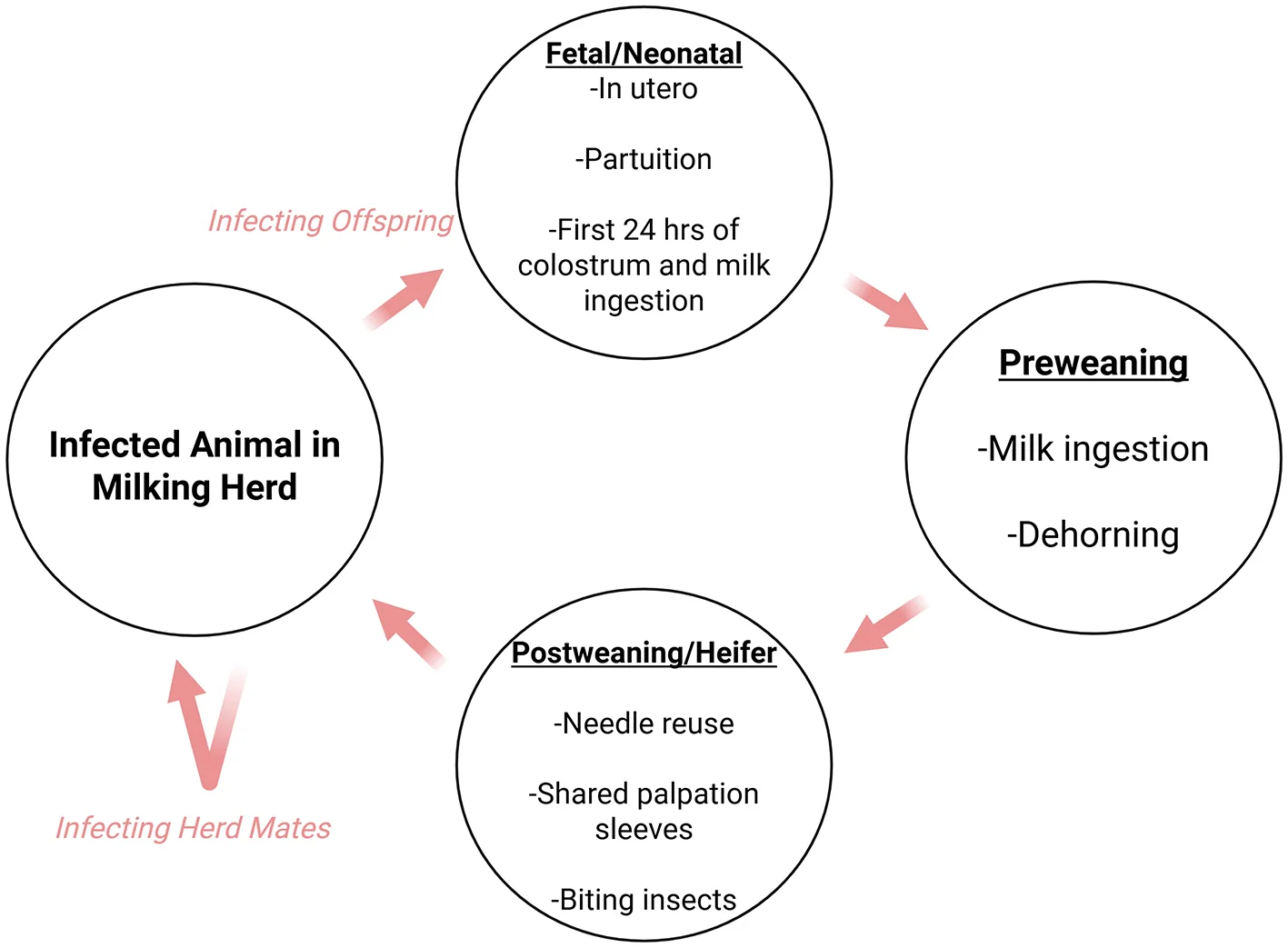
Conceptual cycle of BLV transmission risk across youngstock life stages, showing key risk factors at each stage and pathways by which infected animals entering the milking herd may continue the infection cycle. This figure was created using BioRender.com. Source: Sokacz, M. (2026) https://biorender.com/nuk23z1.

On average, dairy cows break even on the cost of rearing during their second lactation (47, 48) with an estimated average time from first calving to break even of 530 ± 293 days (48). Additionally, there is a significant economic impact of BLV. Infection of cows with BLV results in a partial loss between $198.34 and $496.76 per cow, per lactation (49). If an animal entered the milking herd already infected with BLV, it would likely delay the point at which the animal becomes profitable. A detailed economic analysis or modeling of BLV control in youngstock is beyond the scope of this review; the economic literature is summarized here only to illustrate why early-life infection matters for herd-level control.

## 5 Youngstock time periods

### 5.1 Fetal and neonatal

The fetal stage spans from organogenesis to birth, while the neonatal period is defined as birth through the first 28 days of life (50). Reported fetal BLV prevalence varies, with detection of fetal infections from 0% to 18% (51–53), 6.4–8.1% by AGID (54), or 20% by AGID (41) (Table 1). In terms of neonatal infections, studies have reported a prevalence of 7.7% (42) and 11.47% (53) by PCR, suggesting limited but detectable presence of BLV in calves shortly after birth.

**Table 1:** Reported BLV prevalence in dairy youngstock by life stage, diagnostic method, sample type, and country or region.

Life stage	Assay	Sample type	Country/region	Reported prevalence	References
Fetal	PCR	Blood	Japan	0%	51
Fetal	Immunofluorescence	Serum	USA	18%	52
Fetal	Nested PCR	Blood	Argentina	11.5%	53
Fetal	AGID	Serum	Canada	6.4–8.1%	54
Fetal	AGID	Serum	Japan	20%	41
Neonatal	Nested PCR	Blood	Japan	7.7%	42
Neonatal	Nested PCR	Blood	Argentina	11.47%	53
Preweaning	qPCR	Blood	Japan	0%	77
Preweaning	Qpcr	Blood	Canada	32.1%	78
Preweaning	AGID	Serum	Canada	78%	79
Post-weaning	AGID	Serum	USA	4%	80
Post-weaning	ELISA	Serum	Brazil	38.7%	85
Post-weaning	ELISA	Serum	Uruguay	73.6%	86

Values come from individual studies with differing designs, herd populations, and settings and should not be directly compared across diagnostic methods or studies.

The first risk of BLV transmission that youngstock may encounter begins with the dam and sire. The BLV transfer during gestation was confirmed by the cesarean delivery of youngstock from BLV positive dams (43). In this study, the cord blood, placenta, and peripheral blood of both the dam and calf tested positive for BLV via qPCR and had 100% nucleotide identity with the gene *env*-sequence, which is responsible for virus-cell interaction, between dam and calf, indicating identical strains of BLV between them. An in-depth discussion of the classical genes carried by BLV is beyond the scope of this review; see (55) for further discussion on the BLV *env*-sequence. Additionally, intrauterine infections have been positively correlated with the PVL of the dam (42, 43). Other factors, such as the age, breed, or parity of the dam, were not associated with the likelihood of intrauterine BLV infection (54). Based on previous studies that indicate dams with high PVL have an increased risk of infecting their fetus, BLV qPCR testing of the dam could be prioritized over BLV ELISA testing if breeding considerations are being made based on dam BLV status (42, 43). The impact of sires on the potential of vertical transmission risk has been studied. One hundred twenty-one beef bulls from 39 herds ≥ 2 years of age were sampled and BLV provirus DNA was detected in blood (44.6%) and smegma (7.4%) samples, but not in the semen (56). A more recent study did detect one positive seminal sample via semi-nested PCR (*n* = 1/498) (57). However, at a 30-day follow-up, that same bull's semen tested negative. It is estimated that 89% of dairy operations use artificial insemination for breeding (58) and given the low PCR positivity rate of semen, semen used for artificial insemination is unlikely to be an important source of infection.

Parturition is another potential point of BLV exposure for youngstock. The previous mention of *in utero* transfer was confirmed through the utilization of a cesarean section (43). However, during natural births without surgical assistance, it can be difficult to distinguish between a youngstock born that was infected *in utero* or during parturition. One study aimed to distinguish this by understanding the delay between initial infection and when an assay would be able to detect it. Acknowledging this delay, researchers tested calves 30 days post birth and estimated that 7.7% of youngstock were infected through the vaginal canal (42). Despite the lack of ability to intervene to prevent BLV exposure during the birthing process, BLV can be present within the blood during birth. So, the immediate removal of the calf after birth to avoid further transfer of any fluids, such as cord blood, may reduce risk.

The ingestion of infected colostrum or milk is another well-known exposure risk that youngstock may encounter in the neonatal period, most notably within the first 24 h of life as the neonatal gut allows for the direct absorption of immunoglobulins and cells (59). The impact that colostrum and milk have on BLV transmission is unclear as mammary secretions contain antibodies that may neutralize infections (60–62) but also may contain infected cells that could infect youngstock (63, 64). A study found a positive correlation between PVL of a dam's blood and the PVL in the dam's milk (65). Therefore, there may be a greater transmission risk in the feeding of colostrum and milk from infected dams with higher PVL. Studies focused on calf infections from ingesting infected milk and colostrum have been reported. Study treatments included using unaltered milk and colostrum from BLV positive cows (63, 66) or utilizing both altered and unaltered colostrum (67). Additionally, other studies isolated infected cells from BLV positive cows and resuspended those into milk and colostrum (38, 66, 68) while an early study inoculated sheep using a cultured virus placed in milk as a model (69). Collectively, these studies showed evidence that dairy neonates can be infected through the ingestion of colostrum and milk. However, despite evidence that BLV infections may occur through the ingestion of milk or colostrum, multiple studies have also shown evidence that milk or colostrum could provide antibody protection. A few studies have shown that colostrum from a BLV positive dam, when fed to calves and sheep, created temporary passive immunity (52, 53, 64) that may provide protection against infections for the following 4–6 months (70). Furthermore, a challenge study fed sheep colostrum with anti-BLV antibodies and upon inoculation, the sheep did not become infected (71). A critical part of the treatment of colostrum and milk is the proper treatment to inactivate the virus but still preserve the beneficial components. When using pasteurization to inactivate BLV, the main immunoglobulin found in colostrum and milk, immunoglobulin G (IgG), may be destroyed if not heat-treated properly, resulting in decreased IgG levels (72–74). When utilizing freezing as a method of colostrum and milk treatment, proper thawing protocols need to be taken to reduce the risk of the denaturation of the IgG molecules (75, 76). Given this conflicting evidence, colostrum and milk should not be treated as a uniform transmission risk. These inconsistencies highlight a gap in the current literature that limits the ability to issue definitive management recommendations for colostrum and milk handling.

### 5.2 Preweaning

Dairy youngstock are considered to be in the preweaning stage from approximately 4 weeks of age until weaning at about 8 weeks of age. The prevalence of BLV in preweaning youngstock has a large range and has been estimated as 0% to 32% by qPCR (77, 78) and 78% by AGID (79) (Table 1).

Within the preweaning period, youngstock are mostly reliant on a milk diet, so the risk of BLV transmission via milk ingestion is still applicable. Despite conflicting research between milk's infectivity and its antibody protection, proper freezing or pasteurization should prevent infection and allow the preweaned youngstock to receive antibody protection.

In the preweaning period, youngstock are also susceptible to BLV horizontal transmission. One horizontal transmission risk to consider is dehorning, specifically gouge dehorning. The dehorning process entails the removal of the horn after the animal has formed the horn bud. To accomplish this removal, equipment such as shears, knives, saws, and spoons may be used. Early studies showed that improper or lack of dehorning equipment disinfection between animals resulted in significantly more animals becoming infected (80, 81). An association of transmission was also discovered when gouge dehorning occurred, especially in animals that were dehorned after a known BLV positive animal (82). More recent studies have performed risk factor analyses and found that gouge dehorning practices were associated with a significant increase in overall herd BLV prevalence (29, 83). To prevent BLV transmission through the dehorning process, proper disinfecting of equipment is crucial (84). It should be noted that the use of gouge dehorning in the United States is the least common method among dairy producers (13%) (58). More common methods, such as hot iron (70%) and caustic paste (16%) minimize blood transfer which minimizes the risk of spreading BLV via equipment (58).

### 5.3 Post-weaning

Dairy youngstock are typically weaned from a mostly milk diet to a grain and (or) forage diet by around 60 days of age. The post-weaning period spans from weaning to first calving, during which youngstock become known as heifers. In the post-weaning period, estimates of BLV prevalence have been reported to range from 4% to 73.6% (80, 85, 86) (Table 1). In the heifer period, youngstock may receive vaccinations to combat endemic pathogens (87) or breeding synchronization injections (88–90) depending on the farm management practices. Many of these vaccinations or injections require the use of needles, and the reuse of needles between animals has been repeatedly shown to spread BLV (40, 91, 92) and be positively associated with higher herd BLV prevalence (83, 93). It was estimated that the risk of becoming infected with BLV was eight times greater when animals were sampled for blood using a needle previously used on a known BLV positive herd mate, compared to a known BLV negative herd mate (94). In contrast, other studies have been unable to confirm transmission of BLV through needle reuse (95, 96). Although studies that examine BLV transmission risk through the reuse of needles typically use mature milking animals, the principle of transmission remains the same for youngstock. To prevent the potential horizontal transmission of BLV, single use of needles may be helpful.

Rectal palpations are often utilized on dairies during artificial insemination (AI), pregnancy diagnoses, and monitoring of pregnancies. While sometimes undetectable, veterinarians are aware that animals may experience some degree of hemorrhaging during palpations (97). An initial study confirmed that infection via rectal tissue was possible through the infusion of blood from known BLV positive animals to known BLV negative animals (98). A controlled study aimed to assess transmission risk by using common obstetric sleeves in a known BLV negative animal following the use in known BLV positive animal. The results indicated that when a common obstetric sleeve was used, animals were 8.3 times more likely to seroconvert compared to cases where a new obstetric sleeve was used (99). In contrast, one study was unable to detect new infections through palpation practices (100). The differing results between studies may point to differences in the extent of damage that palpations may cause. Heifers receiving their first rectal palpations are more likely to hemorrhage, so additional observation and caution should be implemented if not using a new palpation sleeve for every animal.

Insects that feed on blood have also been implicated in the transmission of BLV with a study showing a link between biting insect density and BLV infection rate (101). Furthermore, flies collected from dairy farms have been shown to contain both cattle and BLV DNA (102). Biting insects are more abundant in warmer seasons, leading to the theory that BLV infection rates may also be higher in those seasons. However, recorded seasonality of BLV infections is not consistent. For example, BLV incidence was found to be higher in seronegative cattle exposed to seropositive cattle during summer, which is when biting insect populations peak, compared to winter months (103). Contrary to this, a study observed that BLV incidence did not significantly differ between the peak fly season (June through September) and other seasons (104). Additionally, it has been reported that BLV incidence was higher in cattle housed indoors during winter, despite limited exposure to biting insects (105). A controlled study found BLV transmission can occur by moving biting insects from a known BLV positive animal to a known BLV negative animal (106). However, another study found that BLV negative animals housed with BLV positive animals, considered to have persistent lymphocytosis, in the constant presence of biting insects over 4 months, did not become infected (107). Despite the inconsistent findings, insect management is a reasonable and low-risk precaution to consider as part of a broader BLV control strategy. The mixed evidence in both areas underscores the need for more standardized study designs to produce generalizable conclusions. It should be noted that youngstock may be subjected to biting insects throughout the entire youngstock period, not exclusively post-weaning.

## 6 Discussion

This narrative review illustrates the multifaceted nature of BLV transmission and infection in dairy youngstock, emphasizing that infection may happen long before animals enter the milking herd. While many of these risks have been studied individually, their cumulative effect across the early life stages underscores the need for a more comprehensive and proactive approach to BLV control in youngstock. The diagnostics for testing BLV have evolved to allow for more accurate detection and monitoring of infection. The use of ELISA, PCR, and qPCR assays has enabled researchers and producers to assess both antibody presence and proviral burden with greater accuracy. It is evident that management practices, such as reusing needles, mechanical dehorning without disinfection, and palpation sleeve reuse, continue to pose significant risks for horizontal transmission, although not all studies agree. Additionally, while the risk level for vector-borne transmission through biting insects remains unknown, the experimental evidence supports their potential role in spreading the virus, warranting attention to biting insect control in BLV management plans. Economically, the long-term impact of early infection is likely substantial. The raising of heifer replacements already carries a financial burden (47, 48) and, although the actual risk of infection with BLV until heifers' first calving is uncertain, mitigation to reduce the risk of infection of heifers should be part of a whole-herd approach to BLV control. Early detection of BLV infection in youngstock may help diminish economic loss and decrease the overall cost of BLV in any BLV infected herd. However, current evidence is insufficient to support a standardized testing algorithm for youngstock. Age-specific test performance remains poorly characterized, and maternally derived antibodies can confound serologic results in young calves. Until such data are available, testing strategies for youngstock will need to be tailored to herd goals, prevalence, and resources. While mature cows remain central to the conversation of BLV mitigation and control due to their profitable role on a dairy, the failure to address risk of BLV transmission early in life may undermine even the most diligent mature cow-focused control programs. Table 2 summarizes the control measures reported in the literature across these life stages.

**Table 2:** Summary of control measures reported in the literature to reduce BLV transmission risk in dairy youngstock, organized by life stage.

Life stage	Recommended control measure	Reference(s)
Fetal/Neonatal	Prioritize qPCR (PVL) testing of dams over ELISA when making breeding decisions based on BLV status	42, 43
Fetal/Neonatal	Immediate removal of the calf after birth to limit exposure to blood and fluids during parturition	42
Fetal/Neonatal	Proper pasteurization or freezing/thawing of colostrum and milk to inactivate virus while preserving IgG	72–76
Preweaning	Proper freezing or pasteurization of milk fed to preweaned youngstock	72–76
Preweaning	Disinfect dehorning equipment between animals; consider hot-iron or caustic paste methods over gouge dehorning	80–84
Post-weaning	Single use of needles for vaccinations and breeding-related injections	40, 91–94
Post-weaning	Use of new obstetric/palpation sleeves for each animal	98, 99
Post-weaning	Implement biting insect management as a general precaution	101–107

## 7 Review approach

Relevant literature was identified through searches of PubMed and CAB Abstracts, supplemented by manual review of reference lists from key articles. Search terms included combinations of “bovine leukemia virus,” “BLV,” “transmission,” “youngstock,” “calf,” “heifer,” “colostrum,” and “vertical transmission,” with no restriction on publication date due to the historical nature of the BLV literature. Studies were included if they reported on BLV transmission routes, diagnostic methods, or prevalence in youngstock (less than approximately 24 months of age). Studies that focused exclusively on lactating cows were excluded.

This review draws primarily on literature describing dairy production systems in the United States, reflecting the authors' research context. Data from other regions are incorporated where available but remain more limited. Due to this, this review is not intended as a comprehensive global review of BLV epidemiology in youngstock.
